# Modeling juvenile sea turtle bycatch risk in commercial and recreational fisheries

**DOI:** 10.1016/j.isci.2023.105977

**Published:** 2023-01-13

**Authors:** Nathan F. Putman, Paul M. Richards, Susan G. Dufault, Elizabeth Scott-Dention, Kevin McCarthy, R. Taylor Beyea, Charles W. Caillouet, William D. Heyman, Erin E. Seney, Katherine L. Mansfield, Benny J. Gallaway

**Affiliations:** 1 LGL Ecological Research Associates, Bryan, TX 77802, USA; 2 NOAA National Marine Fisheries Service, Southeast Fisheries Science Center, Miami, FL 33149, USA; 3 NOAA National Marine Fisheries Service, Southeast Fisheries Science Center, Galveston, TX 77551, USA; 4 Independent Researcher, Montgomery, TX 77356, USA; 5 Marine Turtle Research Group, Department of Biology, University of Central Florida, Orlando, FL 32816, USA

**Keywords:** Environmental science, Nature conservation, Ecology, Biological sciences

## Abstract

Understanding the drivers of fisheries bycatch is essential for limiting its impacts on vulnerable species. Here we present a model to estimate the relative magnitude of sea turtle bycatch in major coastal fisheries across the southeastern US based on spatiotemporal variation in fishing effort and the simulated distributions of juvenile Kemp’s ridley (*Lepidochelys kempii*) and green (*Chelonia mydas*) sea turtles recruiting from oceanic to nearshore habitats. Over the period modeled (1996–2017), bycatch in recreational fisheries was estimated to be greater than the sum of bycatch that occurred in commercial fisheries that have historically been considered high risks to turtles (e.g., those using trawls, gillnets, and bottom longlines). Prioritizing engagement with recreational anglers to reduce bycatch could be especially beneficial to sea turtle populations. Applying lessons learned from efforts to protect turtles in commercial fisheries may help meet the challenges that arise from the large, diffuse recreational fishing sector.

## Highlights

• A process-based model estimated juvenile sea turtle bycatch in coastal fisheries
• It accounts for variation in ocean conditions, turtle abundance, and fishing effort
• Model estimates reveal sea turtle bycatch is highest in recreational fisheries
• Engagement with recreational anglers, in addition to commercial fishers, is needed

## Graphical abstract

**Figure:**
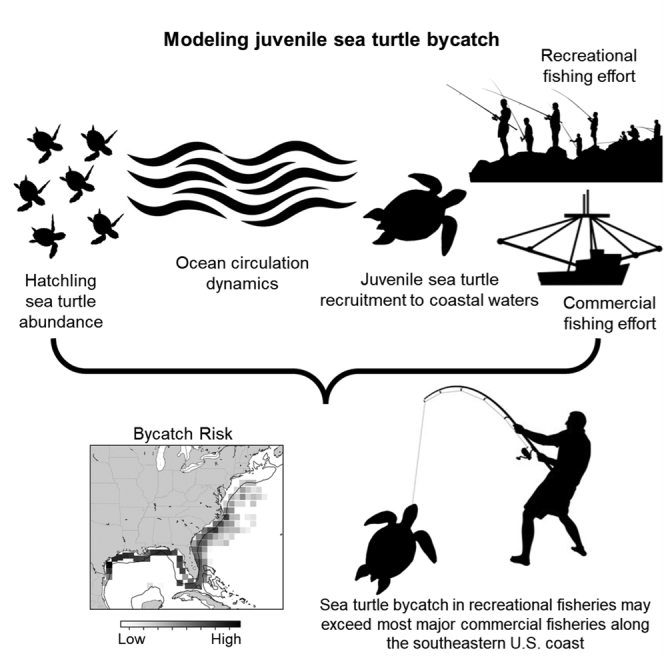

## Introduction

Ontogenetic migration is a common life history trait in marine animals, many of which have an initial dispersive-pelagic oceanic stage followed by recruitment to coastal habitats.^1^^,^^2^ For species such as sea turtles that have relatively low natural mortality rates on reentering coastal habitats, this period of juvenile recruitment is likely important for determining future reproductive potential of the population; high levels of recruitment may directly translate to more mature adults in the future.^3^^,^^4^^,^^5^^,^^6^ Although this ontogenetic migratory behavior allows animals to match environmental conditions with stage-specific physiological requirements, the transition to coastal regions exposes these animals to increased risk from a suite of anthropogenic activities, especially related to fisheries.^1^^,^^7^^,^^8^^,^^9^ The impacts of fisheries bycatch on an individual animal can vary in severity, ranging from mortality to injury and physiological stress. Even sub-lethal effects can reduce the fitness of an individual and, if the bycatch is widespread or of sufficient magnitude locally, can translate to population-level effects.^10^ Bycatch of juvenile turtles during the period of coastal recruitment likely limits a population’s ability to recover from past anthropogenic stressors.^11^^,^^12^

In the US, all sea turtle species are protected under the Endangered Species Act, and bycatch of sea turtles is an important driver in the regulation of commercial fisheries.^13^^,^^14^^,^^15^^,^^16^ Quantifying changes in bycatch through time is often used to determine whether management actions (e.g., gear modifications, time-area closures, or size limits on catch) are succeeding in reducing anthropogenic risks to turtles.^17^^,^^18^ However, bycatch is also affected by the abundance and distribution of sea turtles and the amount and overlap with fishing effort: bycatch is more likely in areas where more turtles are present and where more fishing effort occurs.^16^^,^^19^^,^^20^ The number of oceanic-stage juvenile turtles that recruit into a coastal region in any given year may fluctuate widely because younger age classes tend to be abundant, and their movements are strongly influenced by dynamic ocean conditions.^21^^,^^22^^,^^23^ Annual variability in the abundance and distribution of turtles recruiting to coastal regions can confound the use of fishery-dependent bycatch data to gauge the success of management actions. For instance, is bycatch increasing because mitigative measures are ineffective or because there are more turtles available to be captured?^24^

In this paper, we present a mechanistic approach to better understand the drivers of juvenile Kemp’s ridley (*Lepidochelys kempii*) and green (*Chelonia mydas*) sea turtle bycatch in coastal fisheries across the southeastern US. The model uses spatially explicit fishing effort data and the predicted recruitment of juvenile turtles into those same areas to estimate bycatch. The model does not specify the severity of the bycatch impact (mortality or otherwise), nor does it account for indirect impacts (e.g., lost gear or vessel strikes), rather it identifies the fishing sectors for which the unintentional capture of turtles is most prevalent. This information can contribute to better prioritization of management and engagement programs to reduce turtle interactions in different fisheries.

To model the magnitude of sea turtle bycatch, we assumed that bycatch depends on the spatial overlap between fisheries and turtles and the bycatch rate (catchability) of a particular gear type. To determine the spatial overlap between fisheries and newly recruited juvenile turtles we compiled (1) spatially explicit annual fishing effort data for major coastal fisheries operating in the southeastern US, aggregated by gear type, (2) annual predictions of newly-recruited juvenile turtle abundance within the same spatial areas as the fishing data, and (3) annual observed bycatch within those areas. Further details on these sources of data are available in the STAR Methods. Our approach was to estimate bycatch rates for each fishery as follows:

$$\frac{T_{b}}{T_{p}*F}$$

where T_b_ is the number of turtles observed as bycatch in a given area for a given year, T_p_ is the predicted number of turtles from the model in that area for the same year, and F is the amount of gear-specific fishing effort in the area for that year. This assumed that all bycatch in that area, for that fishery, was observed and is thus the minimum bycatch rate for that area. Locations without observed bycatch were not considered. To estimate the total amount of bycatch we computed the geometric mean of all calculated bycatch rates for a given species-gear type (Table 1). This bycatch rate was multiplied by the total number of predicted turtles in each area multiplied by corresponding fishing effort in each area and summed annually across the southeastern US.

**Table 1:** Estimated bycatch rates of sea turtles in coastal southeastern US fisheries as a function of predicted abundance of turtles and fishing effort

Species	Shrimp trawl (km)	Bottom longline (km ∗ days)	Gillnet (km ∗ days)	Hook & Line (# of lines ∗ days)	Recreational (angler days)
*Kemp’s ridley*	6 × 10 ^−10^ (5 × 10 ^−11^–5 × 10 ^−9^)	5 × 10 ^−10^ (--)	4 × 10 ^−6^ (4 × 10 ^−7^–4 × 10 ^−5^)	–	9 × 10 ^−10^ (7 × 10 ^−11^–2 × 10 ^−8^)
*Green turtle*	5 × 10 ^−12^ (2 × 10 ^−12^–2 × 10 ^−11^)	–	5 × 10 ^−8^ (--)	–	5 × 10 ^−12^ (2 × 10 ^−12^–2 × 10 ^−11^)

Values indicate the geometric mean of all estimates, values in parentheses indicating the geometric mean of computed bycatch rates less than (lower bound) or greater than (upper bound) the geometric mean of all computed bycatch rates. Higher values indicate greater risk (i.e., catchability) to turtles of a given gear/fishery.

## Results

### Fishing effort and juvenile turtle abundance

**Figure 1:**
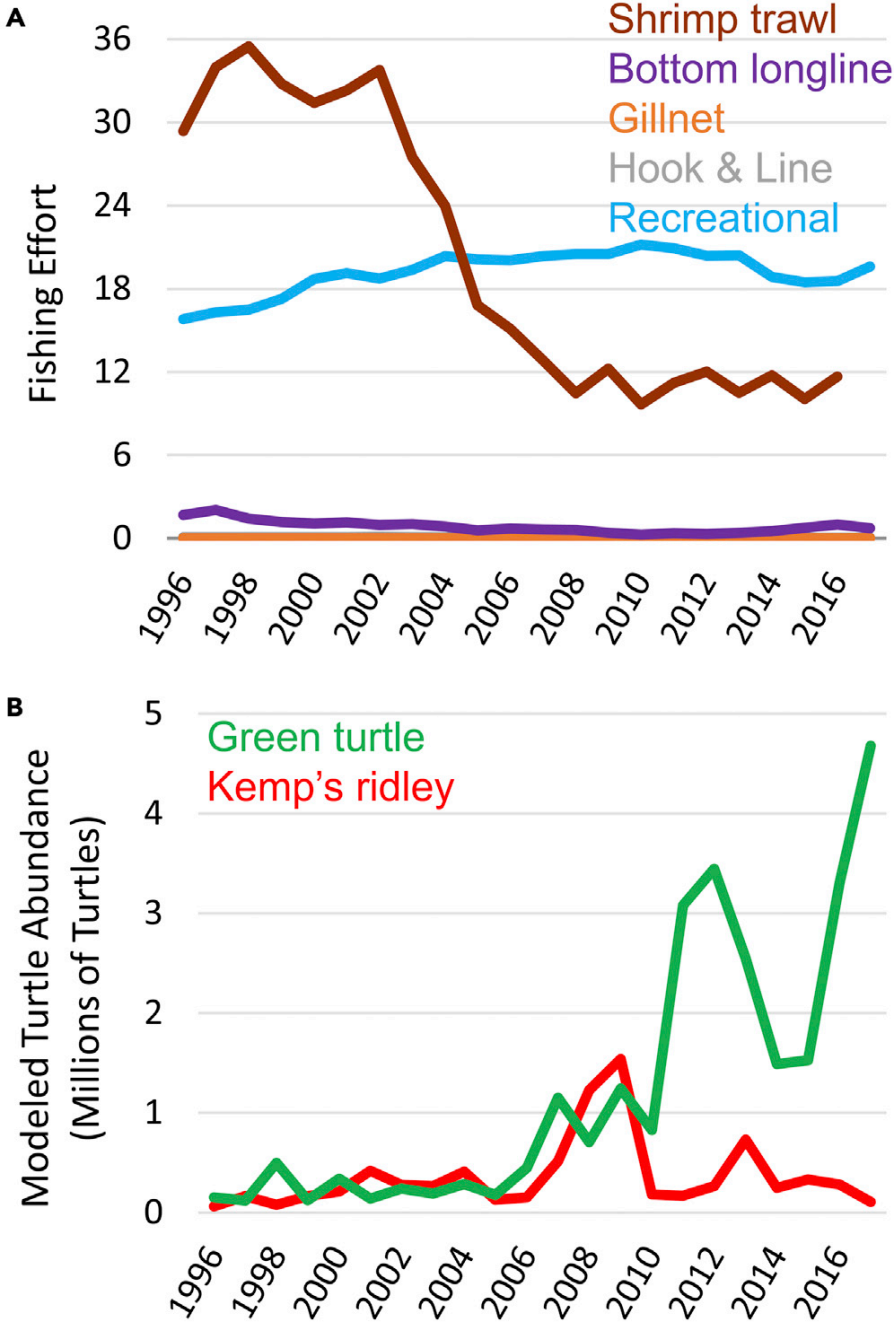
Changes in fishing effort and juvenile turtle abundance (A) Annual fishing effort across the southeastern US from 1996 to 2017 by different fisheries and gear types. Fishing effort units are scaled to reflect differences among gears in the potential for turtle interactions based on the amount of time fishing gear is in the water and the spatial extent of fishing. Shrimping effort (brown line) units are expressed as the total number of days trawled (24 h of actual shrimp trawling by each vessel) multiplied by typical trawling speeds (units = millions of km trawled). Bottom/demersal longlines (purple line) include those targeting sharks, reef fish, and other species. Units are expressed as the average length of a set multiplied by the number of sets and typical soak-time (units = millions of km∗days). Gillnet fisheries (orange line) include drift, run, stake, and others. Units are expressed in the average length of a set (in km) multiplied by the number of sets and the soak-time (units = millions of km∗days). Hook and Line fisheries (gray line) include handline, bandit rigs (electric/hydraulic reels), and trolling. Units are expressed as the total number of lines multiplied by days fished (units = millions of line∗days). Recreational fishing (blue line) includes all saltwater fishing by private fishers and the for-hire charter fishing sectors (Tables 2 and 3). Units are expressed as the number of anglers multiplied by the state-wide mean duration of fishing (units = millions of fisher∗days). Estimated bycatch rates (and thus risk to sea turtles) are shown in Table 1 of the main text. (B) Modeled annual abundance of juvenile Kemp’s ridley (red line) and green (green line) sea turtles entering coastal waters across the southeastern US during 1996–2017. For green turtles, values represent the total numbers of 0.5, to 3.5-year-old turtles in coastal areas each year. For Kemp’s ridley, up to age 2.5 years is represented (Table 4).

Trends in fishing effort differed by fishery (Figure 1A). Decreases in effort were seen in commercial offshore shrimping (Pearson’s r = −0.90, p < 0.0001), hook and line fishing (Pearson’s r = −0.86, p < 0.0001) and bottom longlines (Pearson’s r = −0.74, p < 0.0001). An increase in effort was seen in commercial gillnet fisheries (Pearson’s r = 0.59, p = 0.004) and in recreational fishing (Pearson’s r = 0.59, p = 0.004) (Figure 1A). Trends in predicted juvenile turtle recruitment differed by species (Figure 1B). Early in the time series, modeled recruitment was relatively low, but increasing reproductive output in Kemp’s ridley and green turtles resulted in predictions reaching more than 1.2 million by 2009. Thereafter, modeled juvenile green turtle abundance continued to grow whereas Kemp’s ridley abundance decreased sharply in 2010 and has since leveled off. An overall increase in modeled juvenile recruitment was seen in green turtles (Pearson’s r = 0.81, p < 0.0001) but not for Kemp’s ridley (Pearson’s r = 0.21, p = 0.35).

**Table 2:** Region-wide percentage of recreational fishing effort occurring “Inshore/from shore” (within bays, estuaries, sounds, or along the shoreline), in “Coastal waters” (<3 nautical miles from shore, except for west Florida and Texas which are <10 nautical miles from shore), and “Offshore” (either 3–200 nautical miles from shore or 10–200 nautical miles for west Florida and Texas)

Fishing Location	Mean Effort (%)	Max. Effort (%)	Min. Effort (%)
Inshore/from shore	85.94	87.98	83.65
Coastal waters (0–3 or 0–10 NM)	8.03	9.81	7.10
Offshore (3–200 or 10–200 NM)	6.02	7.19	4.30

To compute bycatch rates and estimate total bycatch we summed effort from each state across each of these subregions. The mean for the entire period and the maximum/minimum values for any given year (1996–2017) show that effort is heavily weighted to nearshore waters.

**Table 3:** Percentage of annual recreational effort for each state that occurred “Inshore/from shore” (1996–2017)

State	Mean (%)	Max (%)	Min (%)
Texas	94.56	98.36	91.58
Louisiana	94.17	98.57	86.68
Mississippi	95.04	98.27	88.60
Alabama	80.29	87.99	68.45
West Florida	73.44	78.56	64.31
East Florida	88.09	90.08	85.54
Georgia	94.71	97.90	91.87
South Carolina	94.47	97.30	90.22
North Carolina	92.07	94.05	88.65
Virginia	96.50	98.89	91.79

**Table 4:** Mean (minimum, maximum) annual percentage of juvenile turtles recruiting to coastal waters across the US by age class (1996–2017)

Species	0.5 years	1.5 years	2.5 years	3.5 years
Kemp’s ridley	60.2 (22.1–98.8)	32.6 (0.4–76.8)	7.2 (0.0–27.3)	–
Green	38.5 (12.6–95.8)	32.1 (3.6–71.3)	19.2 (0.5–70.1)	10.2 (0.1–52.6)

Lower values with increasing age are generally expected because of time-dependent mortality, likewise, the model reflects earlier recruitment of Kemp’s ridley compared to green turtles that has been documented.^39^^,^^69^ To compute bycatch rates and estimate total bycatch we summed turtle abundance across age classes.

### Relative risk and bycatch

**Figure 2:**
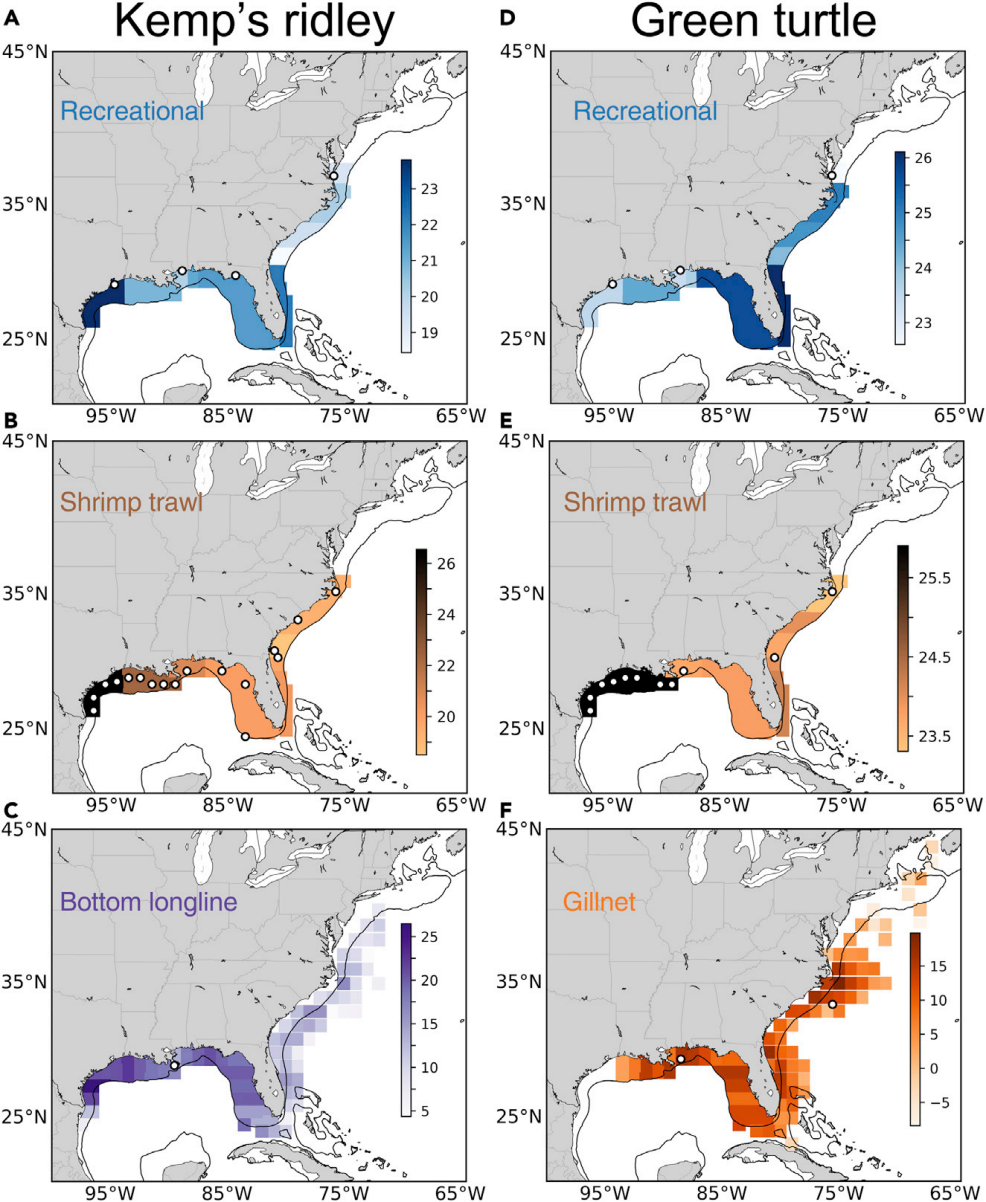
Maps comparing potential overlap between fisheries and juvenile turtles Coloration indicates annual fishing effort multiplied by annual modeled turtle abundance within a given area averaged from 1996 to 2017. Darker colors show regions of higher relative risk (on a log_10_ scale), white circles indicate locations where bycatch was observed. Risk indices for Kemp’s ridley are shown for (A) recreational fishing, (B) shrimp trawling, and (C) bottom longlines; risk indices for green turtles are shown for (D) recreational fishing, (E) shrimp trawling, and (F) gillnets.

**Figure 3:**
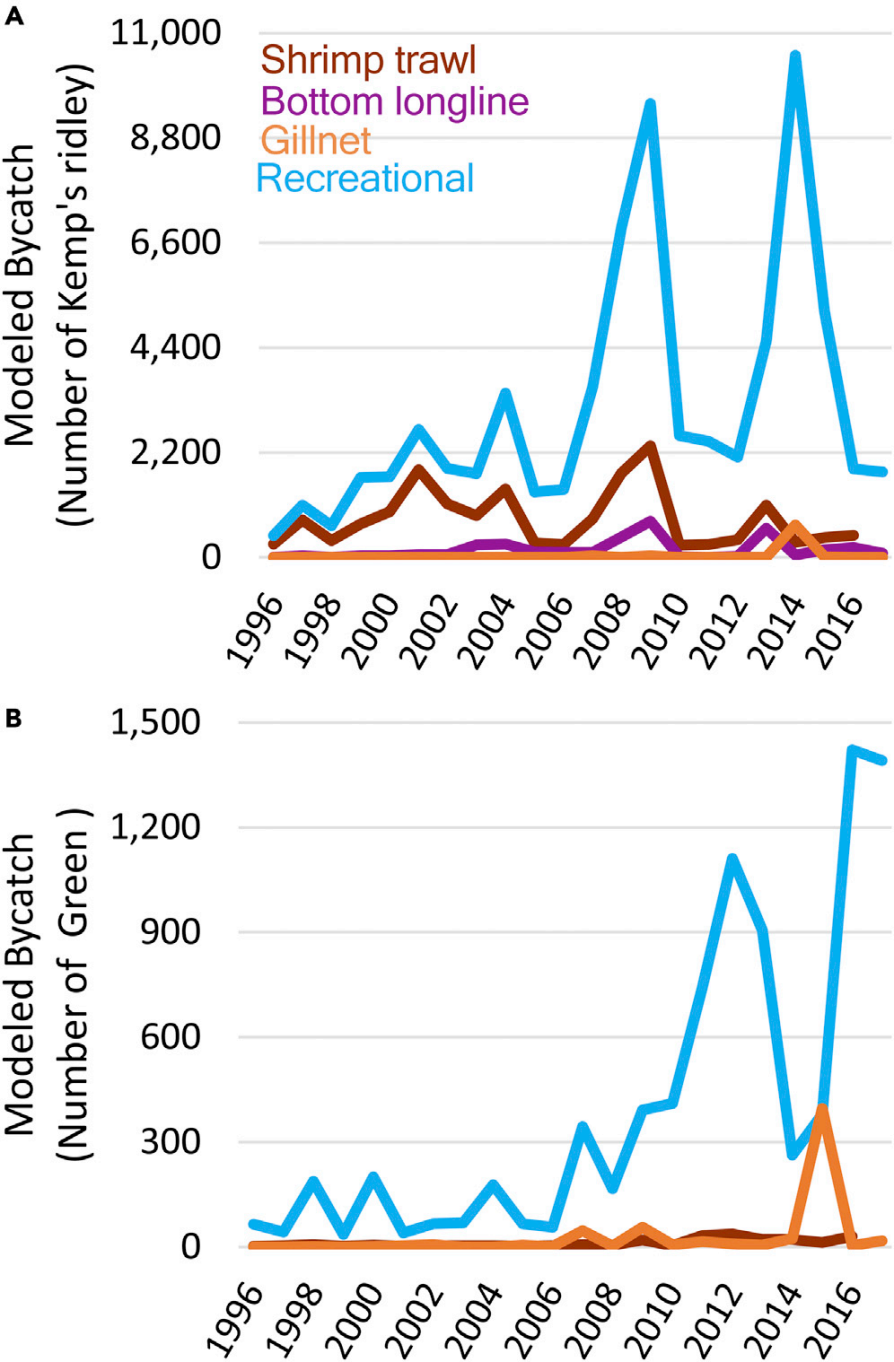
Modeled bycatch by turtle species and fishery/gear type Annual modeled total bycatch for (A) Kemp’s ridley turtles and (B) green turtles based on the spatial overlap between modeled turtle abundance, fishing effort, and calculated bycatch rates (Table 1). Note differences in Y axis scales between species.

The spatial overlap between juvenile sea turtles and fisheries indicated that the potential for interactions differs among species, fisheries, and regions (Figure 2). Calculated bycatch rates for Kemp’s ridley are two orders of magnitude higher than green turtles in shrimp trawls, gillnets, and recreational fisheries (Table 1). Although the magnitude of bycatch rates differs between species, the relative risk of different gears/fisheries demonstrate similarities. For instance, gillnets have the highest bycatch rates by 2–4 orders of magnitude, and a km of shrimp trawling has a similar level of risk to a day of a recreational angler fishing (Table 1). Differences in distribution of turtles and spatial variability in the amount of fishing effort, however, result in the amount of predicted bycatch not being directly proportional to these bycatch rates. For Kemp’s ridley, the geometric mean of annual bycatch was 2,350 turtles in recreational fisheries, 631 turtles in shrimp trawls, 69 turtles in bottom longlines, 1 turtle in gillnets, and no turtles in commercial hook and line fisheries (Figure 3A). For green turtles, the geometric mean of annual bycatch was 203 turtles in recreational fisheries, 7 turtles in shrimp trawls, 4 turtles in gillnets, and no turtles in bottom longline or hook and line fisheries (Figure 3B).

**Figure 4:**
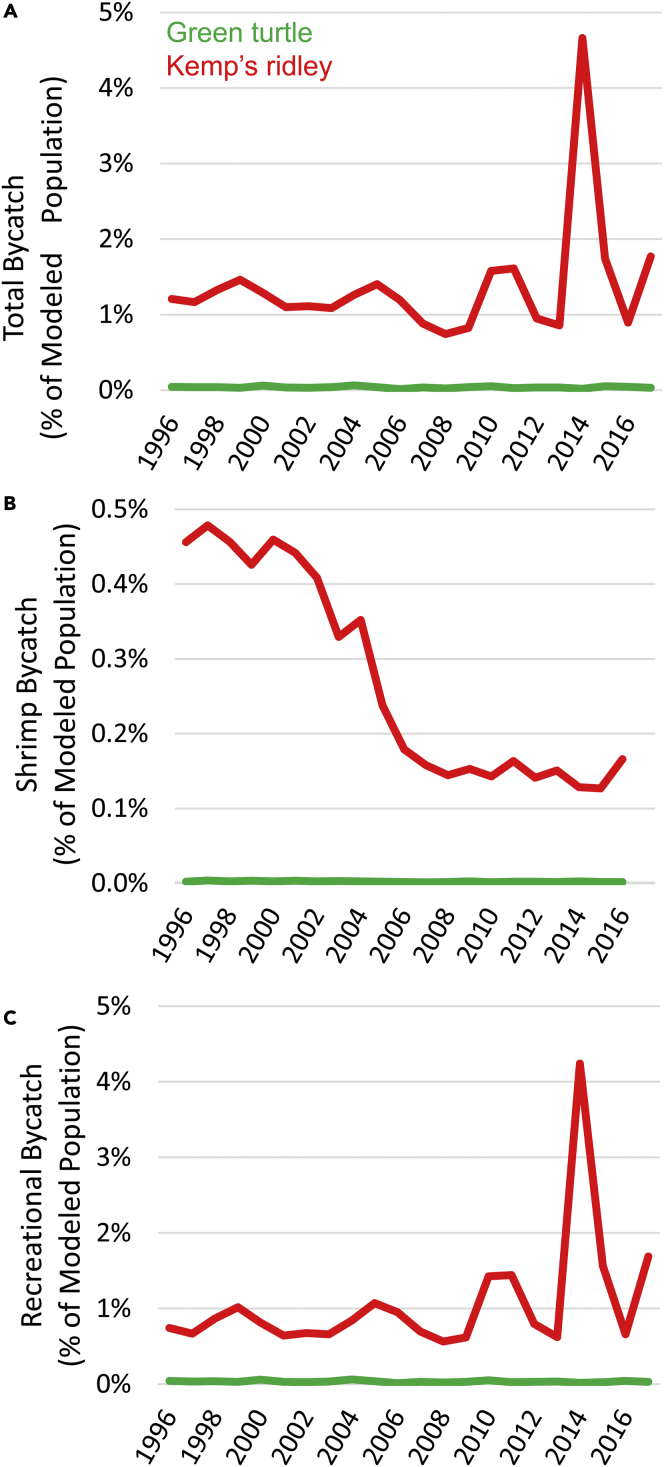
Turtle bycatch as percent of the juveniles recruiting to coastal habitats (A) Total modeled bycatch expressed as the percentage of the modeled population of juvenile turtles for each species (Kemp’s ridley = red, green turtles = green). (B) Shrimp trawl bycatch expressed as the percentage of the modeled population of juvenile turtles for each species. (C) Bycatch in recreational fisheries expressed as the percentage of the modeled population of juvenile turtles for each species. Note differences in Y axis scales among panels.

Predicted bycatch of Kemp’s ridley turtles represents a relatively high portion of the species’ modeled population (ranging from a low of 0.7% in 2008 to a high of 4.7% in 2014) whereas green turtle bycatch is ≤0.06% each year (Figure 4A). Despite temporal fluctuations in modeled population size (Figure 1B) and modeled bycatch (Figure 3), annual total bycatch for all fisheries examined relative to modeled population size appears consistent over the period analyzed (Pearson r < 0.20, p >0.577, n = 22 years, for both species), with the exception of one year for Kemp’s ridley (Figure 4A). Modeled shrimp trawl bycatch relative to turtle population sizes declined through time for Kemp’s ridley (Pearson r = −0.92, p < 0.00001, n = 21 years) and green turtles (Pearson r = −0.68, p = 0.0007, n = 21 years) (Figure 4B). Modeled recreational bycatch relative to turtle population size shows no trend for green turtles (Pearson r = −0.26, p = 0.243, n = 22 years) but has somewhat increased for Kemp’s ridley (Pearson r = 0.413, p = 0.056, n = 22 years) (Figure 4C). Modeled bottom longline bycatch and gillnet bycatch relative to population size show no trend for Kemp’s ridley or green turtles (Pearson r < 0.33, p > 0.133, n =22 years, for each).

### Comparison with other bycatch estimates

**Figure 5:**
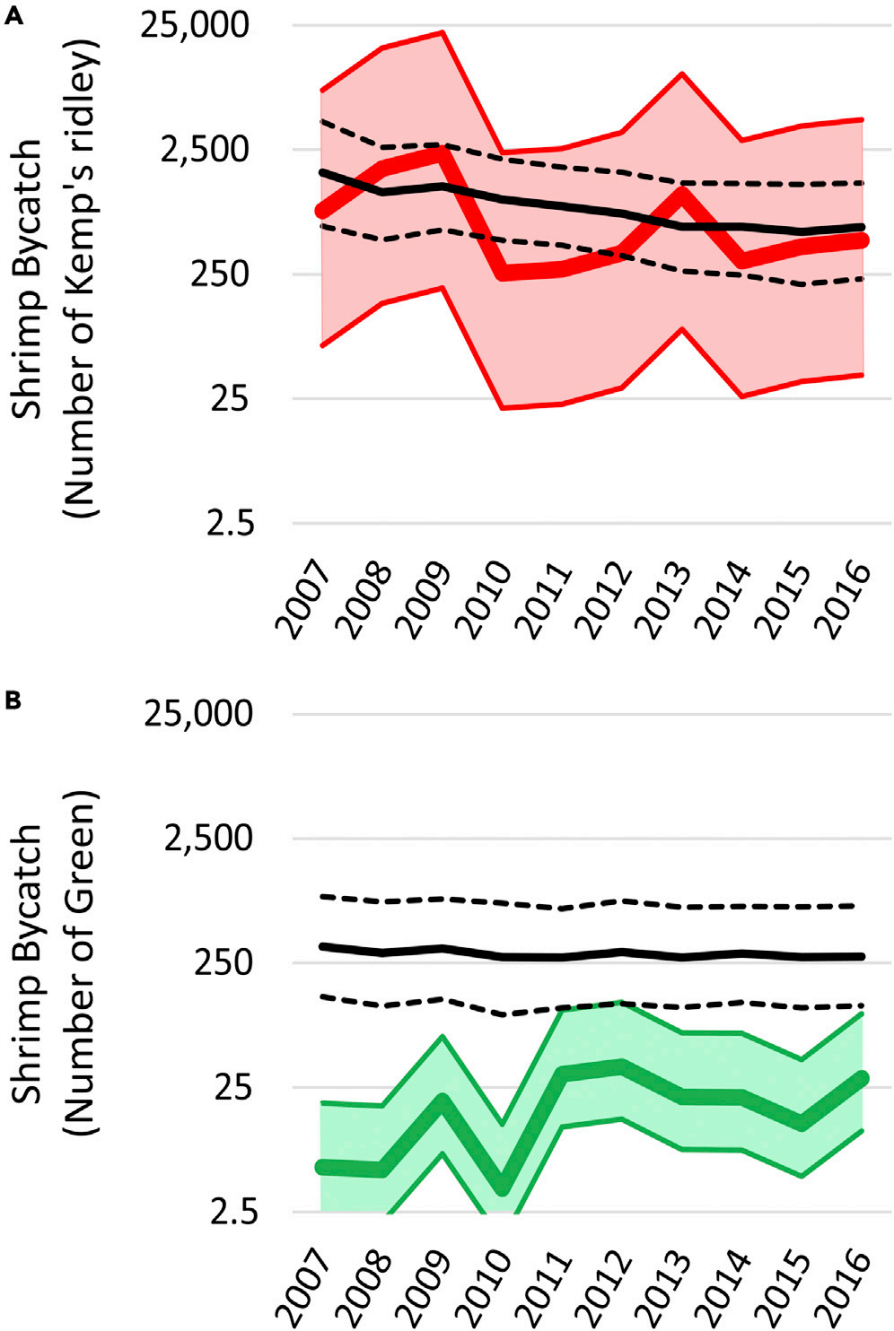
Comparison of modeled bycatch to estimates based on NMFS Observer Programs Colored lines indicate our model of bycatch (thicker lines show the estimate using the geometric mean of all computed shrimp bycatch rates from a given fishery and for a given species. Shading shows the estimates using the geometric mean of computed bycatch rates less than (lower bound) or greater than (upper bound) the geometric mean of all computed bycatch rates). Black lines are published bycatch estimates from NMFS using data from observer programs. Solid lines show the central estimates, dashed lines show the associated uncertainty (95% credible intervals). Estimates of bycatch are shown for (A) Kemp’s ridley and (B) green turtles in the shrimp fishery Note log_10_ scale on y axes.

Bycatch estimates based on National Marine Fisheries Service (NMFS) Observer Programs were available for shrimp trawls (2007–2016). Our model’s predictions of Kemp’s ridley bycatch of 255 to 2,341 turtles per year closely matched observer-based estimates that ranged from 547 to 1,639 turtles per year (Figure 5A). In contrast, our model’s predictions for green turtles were substantially less than shrimp trawl bycatch estimates based on the NMFS Observer Program (Figure 5B). Our model predicted 5 to 35 green turtles, whereas observer-based estimates indicated 227 to 339 green turtles were taken as bycatch.

## Discussion

### Risks from recreational fisheries

Among the most important findings of our synthesis is that recreational fisheries have likely supplanted the federally managed shrimping fleet as the fishing sector with the largest amount of bycatch for Kemp’s ridley and green sea turtles (Figure 3). This finding may seem surprising given that all recovery plans and most syntheses have highlighted shrimp trawling as the largest bycatch threat to sea turtles in US waters with little mention of recreational fishing.^14^^,^^25^^,^^26^^,^^27^^,^^28^ Nonetheless, our model’s prediction is supported by several independent lines of evidence. First, recreational fishing effort has been increasing whereas shrimping effort has declined (Figure 1). In addition, managing shrimp trawl bycatch has received considerable attention and includes NMFS-industry engagement (such as through state Sea Grant offices), gear modification (TED development and rigorous testing), an observer program, and electronic logbooks to monitor effort (in the Gulf of Mexico).^14^^,^^29^^,^^30^^,^^31^ Furthermore, increasing recreational fishing effort is reflected in other related impacts, such as the growing number of registered boats in the state of Florida correlating with increasing boat-strike mortality in sea turtles.^32^ Mean annual estimates of mortality along the Florida coast attributable to boat strikes for Kemp’s ridley (79–316 individuals)^32^ are similar to the mean annual mortality of Kemp’s ridley in shrimp trawls throughout the entire southeastern US between 2007 and 2016 (153 individuals per year).^33^ Annual mortalities in Florida from boat-strikes are much greater for green (505–1,624) turtles^32^ than region-wide shrimping mortalities (green = 58 individuals killed in shrimp trawls per year).^33^ Although not included in our analysis, the same pattern is seen in loggerhead sea turtles (*Caretta caretta*), where boat strikes in Florida are estimated to kill far more loggerheads per year (712–2,292)^32^ than shrimping across the US Gulf and Atlantic coasts (68 individuals per year).^33^

Our model supports the conclusions of other studies that indicate bycatch information in small-scale, coastal fisheries (such as those of recreational fishers) are a problematic data gap, as these fisheries may represent a substantial portion of the total anthropogenic interactions and mortality for sea turtles.^15^^,^^34^^,^^35^^,^^36^ The relatively large potential impact of sea turtle bycatch by recreational fisheries is concerning from a conservation perspective because of the challenges associated with managing a diverse and largely independent group of private fishers.^15^^,^^30^^,^^31^ However, there are encouraging indications of NMFS working to improve outcomes for sea turtles caught on recreational charter vessels by requiring boats to carry tools that can more safely remove hooks from sea turtles.^37^ In addition, private groups have developed programs to educate recreational fishers in how to minimize negative impacts to turtles, for example, the “Responsible Pier Initiative” (https://marinelife.org/conservation/shield/responsible-pier-initiative/). Further stakeholder engagement and research to examine how sea turtle bycatch in recreational fisheries could be reduced and how the rehabilitation of hooked turtles could be improved is recommended.^38^^,^^39^

### Sea turtle ecology and relative bycatch risk

Differences in sea turtle ecology are important for determining the relative risk of bycatch.^40^ Our modeling indicates that although Kemp’s ridley sea turtles are predicted to have lower numerical abundance in the coastal southeastern US than green turtles (Figure 1B), this species has the higher bycatch rates in recreational fisheries (Table 1) and is at the overall highest risk relative to population size (Figure 4A). High bycatch rates of Kemp’s ridley in recreational fisheries are likely because of the turtles’ nearshore distribution where shore-based recreational fishing is concentrated and to a diet that often includes carrion, whereby bait on hooks may be targeted.^41^^,^^42^ Green turtles also aggregate where recreational anglers are concentrated, such as in seagrass beds and along jetties, but their more herbivorous diet likely puts them at less risk.^43^

Modeled bycatch rates in shrimp trawls are higher for Kemp’s ridley than green turtles (Table 1). This corresponds well to the relative risks in shrimp trawls reported by others.^14^^,^^33^ The highest abundances of Kemp’s ridley occur in the western Gulf of Mexico,^23^ which overlaps with the highest levels of shrimping effort.^41^ Green turtles are also abundant in the western Gulf of Mexico;^23^ however, their diet of seagrass and algae^43^ likely results in relatively little time spent over open mud bottoms where they would overlap with the shrimp fishery.

### Limitations of the study

Although this model appears robust for assessing the relative risk to these two sea turtle species in coastal fisheries, several caveats deserve attention. The model (1) does not account for all populations of sea turtles that recruit to US waters, (2) only accounts for the juvenile turtles that have recently recruited from oceanic habitats rather than the entire population, and (3) does not incorporate sea turtle swimming behavior.^22^^,^^23^^,^^44^

Hatchling production inputs differ between the species modeled. For Kemp’s ridley, we have likely accounted for more than 99% of hatchling production for the species (nesting areas in Tamaulipas, Mexico; Veracruz, Mexico; Texas, USA).^22^ For green turtles, annual hatchling production data from potentially important nesting aggregations were unavailable for the years modeled (notably our model does not include the beaches north of Florida, USA; Veracruz, Mexico; Tamaulipas, Mexico; and all nesting areas within the Caribbean, except for Costa Rica). Thus, we expect that our estimates of abundance for Kemp’s ridley should be reasonably accurate, but abundance estimates are likely underestimates for green turtles. Though the model represents upwards of 90% of the green turtle hatchling production of this region, the missing nesting beaches might be expected to contribute up to several hundred thousand additional juvenile turtles to the coastal US in a year.^44^ Regardless, the trends we show in coastal recruits (Figure 1B) generally track trends in nesting abundance of the major populations of both species^45^^,^^46^^,^^47^ and thus are informative for assessing relative risk to these species by fishery.

That this model only accounts for the distribution and abundance of young turtles (<3.5 year old) (Table 4) is likely to give an incomplete picture of bycatch risk. Although there may be indications that the foraging grounds of adult sea turtles could be determined by drift patterns of juveniles^21^^,^^48^ (which would imply our model might indirectly account for adult distributions), explicitly accounting for the numerical abundance, migrations, and habitat use of adult turtles will be a valuable next step.^49^

As noted in the STAR Methods, this model does not include volitional movement by sea turtles to predict distributions but relies on surface currents from an ocean circulation model. The most likely impact of this simplifying assumption is that the abundance of turtles is overestimated in “lower quality” areas and underestimated in “higher quality” areas, as swimming behavior appears to help turtles target favorable regions.^50^^,^^51^ Even so, this model appears appropriate for the broadscale questions posed and has utility for contextualizing atypical events with spatiotemporally explicit predictions of juvenile turtle distributions. For example, the model shows a peak in recreational bycatch of Kemp’s ridley in 2014 (Figure 4), which resulted from a larger portion of the population moving into the Atlantic Ocean and thus being exposed to the relatively high levels of recreational fishing effort that occur on the US east coast. This model peak coincides with the year that the highest number of “cold-stunned” Kemp’s ridley ever was recorded in Massachusetts.^52^^,^^53^ Likewise, the highest number of Kemp’s ridley sightings/strandings in UK waters occurred in 2014 and 2015,^54^ presumably a result of oceanographic conditions that transported many Kemp’s ridley out of the Gulf of Mexico and into the Atlantic Ocean. Nonetheless, this model should be considered a “null hypothesis” of turtle distribution and abundance; additional research to understand how sea turtle behavior contributes to dispersal and recruitment would improve its precision^55^ and allow for finer-scale data on fishing activity and turtle habitat use to address more specific questions on turtle-fishery interactions.^56^^,^^57^

The caveats noted above likely contribute to the discrepancies between modeled bycatch and estimates based on NMFS Observer Program (Figure 5). We expect that not accounting for all turtle populations and life-stages would result in underestimates of the magnitude of bycatch. These two issues were most pronounced for green turtles, and the model predicted an order of magnitude less bycatch than was estimated by NMFS for shrimp trawls (Figures 5B and 5C). In contrast, for Kemp’s ridley we could account for nearly all hatchling production and the age-classes modeled corresponded well the recorded instances of bycatch. The model’s predictions closely matched the estimates produced by NMFS for Kemp’s ridley (Figure 5A), suggesting that when basic input data are available this is a robust approach to estimating bycatch.

### Conclusions

Our model of sea turtle bycatch provides new information on the potential fishery-related threats encountered by turtles recruiting to the coastal waters along the southeastern US. A cohesive and comprehensive strategy to manage bycatch must account for changes in the physical environment,^58^^,^^59^ demographic processes that contribute to differing population trends of various species,^60^^,^^61^ and fluctuations in socioeconomic conditions that influence where and how much fishing effort occurs.^62^ Our model is a step toward that goal and the findings presented can be used to prioritize research and management actions among the fisheries and turtle species examined.

## STAR★Methods

### Key resources table

**Table:** 

REAGENT or RESOURCE	SOURCE	IDENTIFIER
**Deposited data**		

Predicted distributions and abundances of juvenile sea turtles across the Gulf of Mexico and North Atlantic Ocean (1996–2017)	Putman et al.^23^	https://doi.org/10.1111/ecog.04929
NOAA Fisheries’ Marine Recreational Information Program (state-wide estimates of recreational fishing effort 1996–2017)	https://www.fisheries.noaa.gov/recreational-fishing-data/about-marine-recreational-information-program	https://www.fisheries.noaa.gov/data-tools/recreational-fisheries-statistics-queries
NOAA Fisheries' Self-Reported Commercial Coastal Logbook (commercial fishing effort 1996–2017)	Southeast Fisheries Science Center (https://www.fisheries.noaa.gov/about/southeast-fisheries-science-center)	N/A
NOAA Fisheries' Observer Program (sea turtle bycatch records 1996–2017)	Southeast Fisheries Science Center (https://www.fisheries.noaa.gov/about/southeast-fisheries-science-center)	N/A

**Software and algorithms**		

Python version 3.10.5	Python Software Foundation	https://www.python.org

### Resource availability

#### Lead contact

Further information and requests for resources should be directed to and will be fulfilled by the lead contact: Dr. Nathan Putman (nathan.putman@gmail.com).

#### Materials availability

This study did not generate new unique reagents.

### Experimental model and subject details

#### Fishing effort

Fishing effort data were obtained for major coastal fisheries operating in the southeastern U.S. and aggregated by gear type. The spatial extent of effort data differed among data sources and gears. Our aim was to compile spatially-explicit, annual fishing effort data in units that allowed comparison among gear types and fisheries relative to the potential for turtle bycatch based on the amount of time that fishing gear is in the water and the spatial extent of fishing (Figure 1A). All fisheries data spatially overlapped from Texas through North Carolina, but some data extended further north along the eastern U.S. coast. All fisheries data temporally overlapped from 1996 through 2016, but for some fisheries, data were not available for 2017. Data associated with each fishery are described below.

Effort data for shrimp trawls were obtained from National Marine Fisheries Service (NMFS) Galveston Laboratory and Scott-Denton et al.^63^^,^^64^ (for the years 1996–2016), spanning Texas to North Carolina, the areas of the U.S. where nearly all shrimping effort occurs. Gulf of Mexico shrimping effort data were available for the federally managed otter-trawl fishery and were provided as the total number of days trawled per year within four areas that comprised statistical zones 18–21 (∼Texas), 13–17 (∼western Louisiana), 10–12 (∼eastern Louisiana, Mississippi, and Alabama) and 1–9 (western Florida) for the years 1996–2015. Statistical zones are ∼1° in latitude or longitude (depending upon the orientation of the coastline) and extend outward across the continental shelf. Only Gulf-wide effort data were available for 2016 and no data were available for 2017. To partition Gulf-wide effort data to the four areas for 2016, we assumed the relative distribution was the same as in 2015. Atlantic-wide shrimping effort (North Carolina to east Florida) was available for 2007–2016. Shrimp effort in the Atlantic was, on average, 77.5% less than in the Gulf of Mexico (range: 73.0 to 80.3% less). To extend the Atlantic time series of shrimp effort back to 1996, we therefore multiplied annual Gulf of Mexico shrimping effort by 0.225. To spatially partition shrimp effort across the Atlantic we assumed that effort was proportional to the total number of trips taken in statistical zones along the east coast spanning 1° of latitude between 2001 and 2008.^64^ Thus, annual Atlantic shrimping effort was multiplied by 0.085 to obtain shrimping effort for eastern Florida, 0.189 to obtain effort for Georgia, 0.360 to obtain effort for South Carolina, and 0.365 to obtain effort data for North Carolina. While this is inexact, our estimates of sea turtle bycatch were not sensitive to this parameter. In an initial analysis we partitioned Atlantic effort by the average percentage of shrimp trips that were monitored by the NMFS Observer Program between 2011 and 2016.^63^ The proportion of effort was 0.418 in eastern Florida, 0.139 in Georgia, 0.177 in South Carolina, and 0.266 in North Carolina, but estimates of cumulative sea turtle bycatch in shrimp trawls were, essentially, unchanged irrespective of the weighting scheme with a ∼4% difference for Kemp’s ridley and <1% difference for green turtles. For simplicity of presentation, we only report analyses using the total number of trips taken to partition Atlantic shrimping effort rather than those based on the Observer Program’s monitored trips. Shrimp trawling effort was provided as the total number of days trawled (24 hours of actual shrimp trawling by one vessel). We multiplied these values by typical trawling speeds, 2.9 knots (128.9 km/day) in the Gulf of Mexico and 2.6 knots (115.6 km/day) in the Atlantic Ocean^63^ to account for the increased potential exposure of turtles to shrimp trawls as compared to stationary gear.

Data for commercial hook and line, bottom/demersal longlines, and gillnet fisheries were obtained from NMFS Self-Reported Commercial Coastal Logbook (which includes federally managed fisheries, but not those managed by individual states). These data were available from Texas to Maine (1996–2017). Commercial hook and line fisheries include handline, bandit rigs (electric/hydraulic reels), and trolling (895,336 records), but this category does not include for-hire charter fishing. Bottom/demersal longlines include those targeting sharks, reef fish, and other species (63,448 records). Gillnet fisheries include drift, run, stake, and others (43,256 records). We aggregated effort by trip, year, and statistical zone. In the Gulf of Mexico, statistical zones were the same as described for the shrimping effort (∼1° in latitude or longitude along the coastline and extending out to the continental shelf), and in the Atlantic, zones were 1° latitude x 1° longitude. For hook and line fisheries, effort was expressed as the total number of lines reported multiplied by the hours fished (which we converted to days). For bottom/demersal longline fisheries, effort was expressed as the reported average length of a set (in km) multiplied by the reported number of sets and an assumed soak-time of 5 hours (0.2083 days), which is typical in bottom longline fisheries. Gillnet effort was expressed as the reported average length of a set (in km) multiplied by the reported number of sets and the reported soak-time.

Recreational fishing effort was obtained from the NMFS Marine Recreational Information Program (MRIP) query tool for Mississippi, Alabama, Florida, Georgia, South Carolina, North Carolina, and Virginia for 1996–2017. Data for Louisiana were obtained from MRIP for the years 1996–2013 and provided by the Louisiana Department of Wildlife and Fisheries for 2014–2017. Data for Texas recreational fishing were provided by the Texas Parks and Wildlife Department for 1996–2017. Recreational fishing effort estimates from MRIP are statistically extrapolated from survey data to provide the annual number of angler trips in a given state (https://www.fisheries.noaa.gov/recreational-fishing-data/recreational-fishing-data-and-statistics-queries). Effort data included both private fishing and for-hire (charter) fishing, the bulk of which use hook and line gear. These data comprised all saltwater fishing (bays, estuaries, sounds, state territorial seas, and extending seaward 200 nautical miles into the federal Exclusive Economic Zone) within each state. Although recreational fishing effort was aggregated over this entire area to accommodate the broad-scale comparisons of the analyses described below, the distribution of effort is strongly weighted to coastal areas (Tables 2 and 3). Our only modification to these data was to add a temporal component to the effort by multiplying the number of angler trips by the average amount of time fishing in a given state. This information is not provided in the online query tool but can be calculated from the database of survey information that is also available. We calculated average recreational fishing duration to be 5.6 hours in Texas, 4.1 hours in Louisiana, 3.5 hours in Mississippi, 3.4 hours in Alabama, 3.9 hours in western Florida, 3.8 hours in eastern Florida, 3.6 hours in Georgia, 3.0 hours in South Carolina, 2.4 hours in North Carolina, and 3.2 hours in Virginia. As in other fishing sectors, we expressed effort data as days fished.

#### Juvenile sea turtle distribution and abundance

To account for spatiotemporal variation in sea turtle abundance across the southeastern U.S., we used the model outputs from Putman et al.^23^ This model predicts the distribution and abundance of young sea turtles based on annual hatchling production at major nesting beaches, the dispersal of simulated juveniles through the Global Hybrid Coordinate Ocean Model (HYCOM), and stage-specific mortality. The model provides predictions for Kemp’s ridley, green, and loggerhead sea turtles, however, we focus our analyses on Kemp’s ridley and green turtles because the model accounts well for the coastal recruitment dynamics of these species, but not loggerheads.^23^^,^^24^ Three nesting areas were included in the model for Kemp’s ridley: Tamaulipas and Veracruz, Mexico and Texas, USA. Eight nesting areas were included in the model for green turtles: Tortuguero, Costa Rica; Quintana Roo, Yucatan, and Campeche, Mexico; and northwest Florida, southwest Florida, southeast Florida, and northeast Florida, USA. These do not represent all nesting areas but were selected owing to the availability of long-term, consistent monitoring data that allowed consistent indices of annual hatchling production for the years 1993–2017 (the period that ocean circulation model outputs to simulate dispersal were available).

The movement of young sea turtles during their oceanic stage was simulated using Global HYCOM daily snapshots of surface velocity at 0.08° resolution.^65^ HYCOM ocean currents are based on forcing fields and data assimilation that depict ocean conditions at specific times in the past. Dispersal was modeled for years 1993–2017 (HYCOM experiments 19.0, 19.1, 90.9, 91.0, 91.1, 91.2) by ICHTHYOP (ver. 2.2.1) particle-tracking software.^66^ For each nesting region, 350 virtual particles were released daily, offshore of the primary nesting sites during each of the 60 d of peak hatchling emergence. This resulted in 21,000 particles released per region annually for 25 turtle cohorts. ICHTHYOP implemented a Runge–Kutta fourth-order time-stepping method whereby particle position was calculated each half-hour as they moved through the HYCOM velocity fields. Virtual particles were tracked for up to 2.5 yr for Kemp’s ridley and 3.5 years for green. These drift times are representative of the entire oceanic-stage for Kemp’s ridley (∼100% of the oceanic stage) and many green turtles (∼70–100%).

Owing to little empirical data by which to parameterize the model, no attempt is made to simulate swimming behavior of turtles. Several papers show that including swimming behavior in models can influence predicted distributions and associated metrics of ecological relevance.^50^^,^^51^^,^^67^^,^^68^ However, this limitation can be lessened by considering distribution at a relatively broad scale, whereby the influence of fine-scale habitat selection by turtles is reduced and large-scale ocean circulation processes are more important.^44^^,^^55^ With that in mind, model outputs at 1° latitude by 1° longitude resolution were used as an annual index of juvenile recruitment from the oceanic stage to coastal foraging grounds for the years 1996–2017. Particles that occurred within 1° × 1° bins along the U.S. coast were considered “coastal recruits” and the percentage was computed for particles aged 0.5, 1.5, 2.5 and 3.5 yr from each nesting region. For each age class, the percentage of particles was multiplied by the estimate of hatchlings produced at a given nesting region. This value was then multiplied by a daily estimate of oceanic survival based on the median annual estimate (i.e. 81.7%) obtained from the literature.^44^ For each species, these values were summed by age class and nesting region to generate an estimate of turtle abundance in each bin, for each available year (Table 4).

Earlier versions of this modeling approach showed to be excellent predictors of the genetic structure at green turtle foraging grounds along the southeastern U.S. coast,^70^ correspond well to observed ages of Kemp’s ridley recruiting to coastal areas^22^^,^^71^ and closely match abundance estimates based on in-water data of oceanic-stage green, loggerhead, and Kemp’s ridley turtles in the area around the Deepwater Horizon oil spill.^44^ The present implementation of this model shows good agreement in predicting spatiotemporal variation in the recruitment of oceanic-stage green and Kemp’s ridley turtles to coastal waters in the Gulf of Mexico and along the eastern U.S. coast, as inferred from strandings and survey data.^23^^,^^24^ The simulations likewise accurately depict stochastic environmental drivers that contribute to annual variation in turtle distributions.^72^^,^^73^

An important caveat for this model’s use here, however, is that hatchling production inputs differ between the species. For Kemp’s ridley, we have likely depicted upwards of 99% of hatchling production for the species (nesting areas in Tamaulipas, Mexico; Veracruz, Mexico; Texas, USA).^22^ For green turtles, annual hatchling production data from potentially important nesting aggregations were unavailable for the years modeled (notably our model does not include the beaches north of Florida, USA; Veracruz, Mexico; Tamaulipas, Mexico; and all nesting areas within the Caribbean, except for Costa Rica). Thus, we expect that our estimates of abundance for Kemp’s ridley should be reasonably accurate, but abundance estimates are likely underestimates for green turtles. Though we have accounted for upwards of 90% of the green turtle hatchling production of this region, the missing nesting beaches might be expected to contribute up to several hundred thousand additional juvenile turtles to the coastal U.S. in a year.^44^ Regardless, the trends we show in coastal recruits generally track trends in nesting abundance of the major populations of each species^45^^,^^46^^,^^47^ and thus are appropriate for assessing relative risk.

#### Sea turtle bycatch observations

Bycatch depends on the spatial overlap between fisheries and turtles and the catchability of a particular gear type. Our modeling approach implicitly assumes that catchability is proportional to the number of turtles that are observed as bycatch in each gear type and is equal across effort units and habitats for a fishery. Further data, assumptions, and analyses would be needed to explicitly estimate relative catchability by gear type, habitat, and fishery but is outside the scope of this study.

Bycatch in commercial fisheries was determined using NMFS Observer Program records. This provided us with 95 bycatch records in federally-permitted shrimp trawls (2007–2016), 5 bycatch records in gillnet fisheries (2000–2016), 1 bycatch record in bottom/demersal longline fisheries (2003–2017), and 0 bycatch records in hook and line fisheries (2008–2016). For recreational fisheries bycatch, we obtained 1180 bycatch records (1996–2017) from published sources, which were aggregated by state or sub-region of a state.^38^^,^^74^^,^^75^^,^^76^ For much of the observed bycatch data, the sizes of individuals were unavailable. Thus, it was not possible to parse smaller juvenile turtles (i.e., those recently transitioned from oceanic to coastal habitats) from older juveniles and adults. Given that observed bycatch is necessarily an underestimate of actual bycatch we opted to use all bycatch records available, without respect to turtle size (Table S1).

### Method details

To generate bycatch rates for each fishery, we aggregated bycatch annually into 1° × 1° bins that coincided with the turtle distribution and abundance model described above. We then divided the annual amount of bycatch by the product of annual modeled number of turtles and the annual fishing effort in that same bin. To partition shrimping effort and recreational fishing effort into 1° × 1° bins we divided the amount of effort in an area or state by the number of 1° × 1° bins across that part of the coastline (e.g., recreational fishing effort across Texas was divided by 4, whereas recreational fishing effort across Mississippi was not modified). This was conducted for all available instances of bycatch and aggregated by species and gear type. We then computed the geometric mean of all calculated bycatch rates for a given species-gear type (Table 1). We opted to apply the geometric mean of all bycatch rates to limit the influence of particularly high or low bycatch rates and because there was no temporal trend in bycatch rates for any of the datasets (R^2^< 0.17, p > 0.064, n < 49). We extrapolated the total bycatch by multiplying that rate of bycatch (caught turtles/(available turtles ∗ fishing effort)) across the summed annual risk indices (all available turtles ∗ all fishing effort) for the southeastern U.S. Risk indices are shown for the shrimp fishery (Table S2), recreational fisheries (Table S3), bottom longline fisheries (Table S4), gillnet fisheries (Table S5), and hook and line fisheries (Table S6). Our approach for deriving bycatch rates implicitly assumed that all bycatch within an area was observed, but there is little reason to believe this is the case, and our model might be expected to underestimate actual bycatch. However, our approach also does not account for the many fishing trips that did not catch turtles, which could potentially result in overestimating actual bycatch. While the absolute magnitude of bycatch in a given gear type is uncertain, our model captures a relative amount of bycatch risk among gear types. As such, our model is best suited for examining in which fisheries bycatch risk may be greatest and thus where conservation and management effort might be most beneficial to turtles.

### Quantification and statistical analysis

We compared our model’s estimates of bycatch to those based on NMFS Observer Programs for shrimp trawls (2007–2016).^33^ Babcock et al.^33^ represents the most rigorous and robust characterization of sea turtle bycatch in shrimp trawls using observer data to-date and is particularly valuable given the general assumption that shrimp trawl bycatch in U.S. fisheries has an order of magnitude greater impact on turtles than all other fisheries combined.^11^^,^^25^^,^^26^ Babcock et al. used data from the NMFS Observer Program and Bayesian techniques to model Kemp’s ridley, green, and loggerhead turtle bycatch for the U.S. Gulf of Mexico (2007–2015) and Atlantic coast of the U.S. (2007–2016).^33^ The Babcock et al. model divided bycatch into total bycatch and mortalities as well as bycatch that occurred in standard trawl nets and try nets.^33^ Given our interest in total bycatch, we summed the annual total bycatch in both standard nets and try nets in the Gulf of Mexico and Atlantic for the median estimates and 95% credible intervals for each species. Data were unavailable for the Gulf of Mexico in 2016; to present an estimate of total shrimp trawl bycatch for that year we took the mean values over the previous three years to add to the estimates of Atlantic coast bycatch.

For visual comparison of the relative uncertainty associated with the observer-based and model-based methods, we plotted our model’s estimates using the geometric mean of the total sample of computed bycatch rates bracketed by the geometric mean of the values that were greater than (upper limit) or were less than (lower limit) the total geometric mean computed bycatch rates. NMFS-generated bycatch estimates show the median bracketed by the 95% credible intervals.

These NMFS program estimates bycatch using the same data sources as our model; both consider total fishing effort and the number of turtles observed as bycatch. However, observer programs use the proportion of observer coverage to extrapolate bycatch across the fishery but do not account for spatiotemporal variation in the distribution or abundance of turtles. Our model does not account for observer effort, but instead accounts for the distribution and abundance of juvenile turtles to extrapolate bycatch across the fishery, which can be an important driver in changes in bycatch rates.^24^ In short, each approach has a potentially major weakness, but the benefit of our model is that it can generate estimates when there is no information on observer effort, as is the case for recreational fisheries. Our main interest in this comparison is to provide some indication as to whether the magnitude of modeled bycatch represents an overestimate or underestimate from traditional approaches.

#### Description of supplemental spreadsheets

Spreadsheets are available in Microsoft Excel format that show location-specific risk indices of sea turtle bycatch, fishing effort and predicted sea turtle abundance by species, year, and gear type that were referenced in the STAR Methods. The file is named “ DataS1_Star_Methods_Turtle-Bycatch.xlsx” Table S1, “Bycatch”, the year, species, count, and location of observed bycatch is reported for each fishery. Shrimping bycatch is highlighted brown, bottom longline bycatch is highlighted purple, gillnet bycatch is highlighted orange, recreational bycatch is highlighted blue. Table S2, “Shrimp_Risk”, spatial areas are designated as GOM_Area_4 (corresponding approximately to the coastal waters of Texas), GOM_Area_3 (corresponding approximately to the coastal waters of Louisiana), GOM_Area_2 (corresponding approximately to the coastal waters of Mississippi/Alabama), GOM_Area_1 (corresponding approximately to the coastal waters of west Florida), EFL (corresponding approximately to the coastal waters of east Florida), GA (corresponding approximately to the coastal waters of Georgia), SC (corresponding approximately to the coastal waters of South Carolina), and NC (corresponding approximately to the coastal waters of North Carolina). Estimates for Kemp’s ridley’s overlap with shrimping effort are presented first, followed by green turtles. Other tabs follow these same conventions. Table S3, “Rec_Risk”, risk indices between turtle species and recreational fishing effort data are presented by state: TX = Texas, LA = Louisiana, MS = Mississippi, AL = Alabama, WFL = west Florida, EFL = east Florida, GA = Georgia, SC = South Carolina, NC = North Carolina, VA = Virginia. Table S4, “BLL_Risk” (bottom longline fisheries), Table S5, “Gillnet_Risk” (gillnet fisheries), and Table S6, “H&L_Risk (hook and line fisheries), spatial data are presented by NMFS Statistical Zone (∼1° latitude x ∼1° longitude blocks) for the Gulf of Mexico. Gulf of Mexico Statistical Zone 1 (GOM_Z1) corresponds to the Florida Keys. The numbering system continues along the coastal US to the Texas/Mexico border ate Statistical Zone 21 (GOM_Z21). Other columns (Y-FZ) indicate the center point of 1° latitude x 1° longitude blocks, with the first two digits in the column header providing the latitude (°N) and the last two digits providing the longitude (°W).

## Data Availability

All data reported in this paper will be shared by the lead contact upon request. This paper does not report original code. Any additional information required to reanalyze the data reported in this paper is available from the lead contact upon request.
